# The role of chain stretch heterogeneity on the uniaxial failure response of rubbery networks

**DOI:** 10.1016/j.mechmat.2026.105599

**Published:** 2026-04

**Authors:** Lucas Mangas Araujo, Laurence Brassart

**Affiliations:** Department of Engineering Science, University of Oxford, Oxford OX1 3PJ, United Kingdom

**Keywords:** Rubber elasticity, Damage, Chain scission, Micromechanics, Fracture

## Abstract

The macroscopic failure response of rubbery networks such as elastomers and hydrogels involves the scission of individual polymer chains, mediated by the network topology. However, the precise mechanisms by which individual chain scission events result in macroscopic failure remain poorly understood. In this work, we use Discrete Network (DN) simulations to investigate failure mechanisms in model random networks in uniaxial tension. Our DN simulation results suggest that macroscopic failure, characterised by a sharp drop in the macroscopic stress–stretch response, only requires the scission of a small fraction of chains in a localised region of the network, even in perfect monodisperse networks. Localised failure is triggered by pre-existing heterogeneities in the chain stretch and is further modulated by network parameters such as the chain length or chain strength. Simple micromechanical models of rubber elasticity, such as the three-chain and eight-chain models, fail to capture the onset of damage because they do not capture the chain stretch heterogeneity. More sophisticated microsphere theories in their affine and non-affine versions only partially address this shortcoming. Overall, our results provide new insights into failure mechanisms of rubbery networks, while providing reference results useful for the validation of improved constitutive theories.

## Highlights

• Discrete network (DN) simulations of monodisperse and bimodal networks.
• Localised failure is triggered by pre-existing stretch heterogeneity.
• Network failure only requires the scission of a small fraction of chains.
• Systematic comparison between DN simulations and continuum models.
• Extended microsphere models accounting for pre-stretch distribution.

## 1 Introduction

Rubbery networks, such as elastomers and hydrogels, are found in many applications, from daily goods like tyres, seals and footwear to biomedical implants (Caló and Khutoryanskiy, 2015) and soft robotics (Lee et al., 2020). In these materials, the mechanical properties such as stiffness, strength and stretchability are primarily dictated by network characteristics such as the polymer chain length distribution, the crosslink density, and the presence of network heterogeneities and topological defects. Consequently, recent design strategies for soft materials have aimed to achieve superior mechanical properties through careful control of the network structure (Zhao et al., 2021, Li and Gong, 2024). For example, near-ideal tetra-PEG gels were obtained by combining four-arm macromolecules in solution to produce networks with high strength (Sakai et al., 2008, Matsunaga et al., 2009). Tough hydrogels have been obtained by introducing energy-dissipative mechanisms into the network, for example in double-network and hybrid gels (Sun et al., 2012, Gong, 2014, Li and Gong, 2024). Recently, introducing entanglements has been shown to increase resistance to crack growth (Kim et al., 2021, Nian et al., 2025). The introduction of mechanophores in the network has also emerged as an interesting approach to gain insight into the failure mechanisms in rubbery networks at the molecular level (Wang et al., 2021, Beech et al., 2023, Ju et al., 2023) or to improve their toughness (Wang et al., 2023a).

To gain a precise understanding of the role of network parameters on the deformation and failure, micromechanical approaches that can address the length scale of the polymer network are required. In this respect, Discrete Network (DN) models have recently emerged as promising tools, providing a computationally efficient alternative to molecular dynamics simulations while capturing essential network characteristics (Sugimura et al., 2013, Kothari et al., 2018, Alamé and Brassart, 2019). In DN models, the molecular network is represented by an assembly of springs with behaviour derived from statistical mechanics considerations. Unlike in continuum micromechanics models of rubber elasticity (Boyce and Arruda, 2000), the partitioning of forces and stretches among the chains is calculated from the condition of mechanical equilibrium at the crosslinks, rather than being imposed through ad hoc assumptions. As such, DN models constitute a physically grounded and computationally tractable approach to analyse the relationships between single chain behaviour, network structure and macroscopic response of rubberlike materials. In recent years, several studies have used DN models to investigate structure–property relationships in rubbery networks (Wagner et al., 2022, Mangas Araujo et al., 2024, Lei et al., 2025). In the context of fracture, DN models were used to assess the validity of the Lake–Thomas model (Lei and Liu, 2022, Deng et al., 2023, Hartquist et al., 2025b, Hartquist et al., 2025a), and to unveil the microscopic origins of crack paths typically observed in soft materials (Ghareeb and Elbanna, 2020, Lei et al., 2021). Chain scission kinetics has also been included in DN models to describe rate-dependent deformation and failure of rubbery networks (Kothari et al., 2018, Ghareeb and Elbanna, 2021, Arora et al., 2022, Beech et al., 2023, Mangas Araujo and Brassart, 2025).

Despite these recent contributions, the potential of DN simulations to understand the micromechanisms of failure has not yet been fully explored. The main objective of the present work is to investigate these micromechanisms in model networks in uniaxial tension. We show that failure is a heterogeneous process, involving highly localised chain scission events, even in ideal monodisperse networks. We further investigate the role of network heterogeneities on the failure response by considering networks of chains with two different lengths and two different strengths. A second objective of this work is to assess the predictive capability of semi-analytical micromechanical models using DN simulations as reference. We show that commonly-used models based on the three-chain or eight-chain representations of the network fail to predict the onset of failure. This occurs primarily because these models neglect the distribution of chain stretch existing in random networks. To address this limitation, we further investigate affine and fully-relaxed microsphere damage models extended to account for the initial distribution of the chain pre-stretch, building on previous works (Mulderrig et al., 2021, Mangas Araujo et al., 2024). While an extended affine microsphere model accounting for the initial chain stretch distribution yields a good estimate of the peak stress, none of the tested models can simultaneously capture the peak stretch, peak stress and abrupt stress drops observed in DN simulations.

The paper is organised as follows. Section 2 presents the DN methodology. In Section 3, we investigate the failure response of ideal, monodisperse networks. Networks with bimodal distributions of chain length and chain strength are studied in Section 4. Limitations of our DN approach and perspectives for future works are discussed in Section 5, before concluding.

## 2 Discrete network model

The DN modelling framework adopted in this work is essentially the same as in our previous work (Mangas Araujo et al., 2024), the main difference being that we now account for (rate-independent) chain scission. The main elements of the models are provided below. For more details about the theoretical foundations and numerical aspects of the model, we refer to Mangas Araujo et al. (2024).

### 2.1 Chain behaviour

The freely-jointed chain (FJC) model is used to describe the mechanical behaviour of individual chains. The free energy of a chain with end-to-end distance r is given by:

(1) $$w=Nk_{B}T\left[\frac{r}{Nb}\beta +\log\left(\frac{\beta }{\sinh\beta }\right)\right],\;\beta =L^{-1}\left(\frac{r}{Nb}\right),$$

where b is the Kuhn length, N is the number of Kuhn segments, $k_{B}$ is the Boltzmann constant, and T is the absolute temperature. The Langevin function is defined as L(x)=coth(x)−1/x. The force f required to maintain the end-to-end distance r is derived from the free energy as $f=\frac{dw}{dr}$:

(2) $$f=\frac{k_{B}T}{b}L^{-1}\left(\frac{r}{Nb}\right).$$

In the numerical implementation of the model, we use the Padé approximation of the inverse Langevin function to calculate the chain force: $L^{-1}\left(x\right)\approx x\frac{3-x^{2}}{1-x^{2}}$ (Cohen, 1991). For small elongations (r≪Nb), L(x)≈3x and the force–extension relationship Eq. (2) becomes linear, recovering the Gaussian chain model:

(3) $$f=\frac{3k_{B}T}{Nb^{2}}r.$$

The chain pre-stretch is defined as the ratio of the end-to-end distance $r_{0}$ in the initial configuration to the ideal random walk distance $\sqrt{N}b$: $\lambda_{0}=\frac{r_{0}}{\sqrt{N}b}$. Chain scission is assumed to occur when the chain end-to-end distance r reaches a given fraction η of its contour length, i.e. when $\frac{r}{Nb}\geq \eta$.

At large chain elongations, the FJC model is inaccurate as it neglects enthalpic effects due to bond stretching and bond angle deformation. In principle, these effects can be incorporated in the current DN framework by adopting more sophisticated chain models that account for stretchable Kuhn segments (Smith et al., 1996, Mao et al., 2017, Guo and Zaïri, 2021, Mulderrig et al., 2023) or deformable bond lengths and bond angles (Lavoie et al., 2020, Zhu and Brassart, 2025). In this work, the classical FJC model is retained for simplicity, and also to facilitate the comparison between DN predictions and analytical estimates.

### 2.2 Network behaviour

We consider a network of freely-jointed chains interacting with each other via their common crosslink points, i.e. we assume a phantom network. In the DN model, polymer chains are represented as springs connected at nodes, corresponding to the crosslinks. The elastic energy density of the network is given by:

(4) $$\psi^{e}=\frac{1}{V_{0}}\overset{n}{\underset{j=1}{\sum }}w_{j}\left(r_{j},N_{j}\right),$$

where $V_{0}$ is the volume of the network, $w_{j}$, $r_{j}$ and $N_{j}$ are the free energy, end-to-end distance and number of Kuhn segments of the jth chain, respectively, and n represents the current number of elastically-effective chains in the network. This number can change during deformation due to chain scission. The macroscopic deformation is applied to the network by prescribing the displacement of $n_{b}$ boundary nodes according to the affine relation:

(5) $$x_{\alpha }=FX_{\alpha },\;\alpha =\left(1,n_{b}\right),$$

where $X_{\alpha }$ and $x_{\alpha }$ represent the position of the αth boundary node in the reference (initial) and current configurations, respectively, and F is the macroscopic deformation gradient. In the reference configuration, F=1. For given positions of the boundary nodes, the position of the $n_{i}$ interior nodes is found by minimising the elastic energy of the network (4), corresponding to the condition of force balance at each crosslink. The elastic contribution to the macroscopic nominal stress is obtained as (Alamé and Brassart, 2020):

(6) $$P^{e}=\frac{1}{V_{0}}\overset{n_{b}}{\underset{\alpha =1}{\sum }}f_{\alpha }⊗X_{\alpha },$$

where $f_{\alpha }$ is the reaction force at boundary node α. It can further be shown that $P^{e}=\frac{\partial \psi^{e}}{\partial F}$. Assuming incompressibility of the network (J=det(F)=1), the total nominal stress is given by:

(7) $$P=P^{e}-JPF^{-T},$$

where P is a Lagrange multiplier identified from the boundary conditions. The Cauchy stress is obtained from the classical relation: $\sigma =\left(1/J\right)PF^{T}$, giving:

(8) $$\sigma =\sigma^{e}-P1,$$

with $\sigma^{e}=\left(1/J\right)P^{e}F^{T}$.

According to the FJC model (2), the chain force is zero only when the chain end-to-end distance is zero, corresponding to its coiled configuration. It follows that reaction forces must be applied to the boundary nodes in the reference configuration to achieve a non-zero initial volume $V_{0}$ and prevent network collapse. This means that $P^{e}$ does not vanish in the reference configuration. Assuming that the network is isotropic and initially stress-free, the Lagrange multiplier is identified from Eq. (8) as $P=\frac{1}{3}\mathrm{tr}\left(\sigma^{e}\right)$ in the reference configuration.

### 2.3 Numerical procedure

**Fig. 1:**
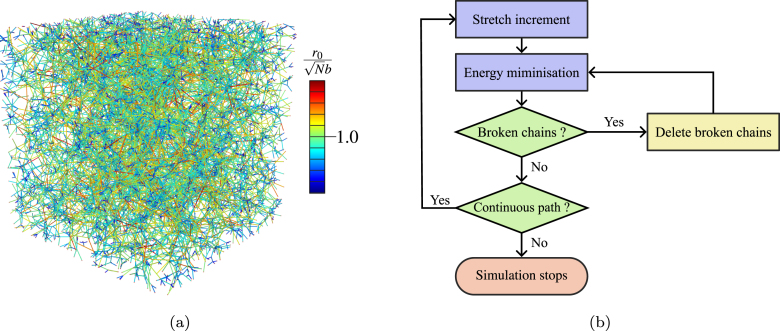
(a) Representative discrete network with n=20000 chains in the equilibrated reference configuration, showing the initially heterogeneous distribution of chain end-to-end distances. (b) Numerical procedure for the simulation of discrete networks up to loss of connectivity.

Discrete networks were generated in cubic domains of volume $V_{0}$ using our in-house network generation algorithm implemented in MATLAB. This algorithm allows for independent control over chain density and end-to-end distance distribution by allowing the chains to interpenetrate, see Mangas Araujo et al. (2024) for details. The coordination of the crosslinks was four, and networks did not contain any dangling ends or loops (i.e. they were perfect networks). As-generated DNs contained at least 50 000 chains, which is sufficiently large to ensure that the networks are statistically representative and isotropic. For each set of input parameters, five independent network realisations were generated to capture variability in failure behaviour. A representative network is shown in Fig. 1(a).

We consider networks subjected to macroscopic deformation gradients of the form $F=\mathrm{diag}\left(\lambda_{1},\lambda_{2},\lambda_{3}\right)$, where $\lambda_{i}$ (i=1,2,3) are the principal stretches along the faces of the cubic domain. We limit ourselves to uniaxial tension along direction 1, which implies that $\lambda_{2}=\lambda_{3}=\lambda_{1}^{-0.5}$ from the incompressibility constraint. At each time step, a stretch increment $\Delta \lambda_{1}=0.04$ was applied. We have verified that this stretch increment is sufficiently small so that the deformation and failure response remains unchanged for smaller values of $\Delta \lambda_{1}$. Since our model is rate-independent, we do not specify a physical time step. During a given macroscopic stretch increment, the boundary nodes were kept fixed while the elastic energy of the network was minimised using LAMMPS (Thompson et al., 2022). After minimisation, the network was scanned for broken chains. If no chains met the breaking criterion, the simulation proceeded to the next time step. However, if some chains were found to have reached their scission threshold, these chains were removed from the network, and the system was re-equilibrated by energy minimisation. This scission detection and re-equilibration loop was repeated until no further chain scission was detected in a given time increment. A similar procedure was adopted in other recent works (Ghareeb and Elbanna, 2021, Lei et al., 2021, Lavoie and Suo, 2024, Lei et al., 2025). Before proceeding to the next time step, the network was checked for loss of connectivity, defined as the loss of a continuous path connecting the opposite faces of the simulation box in the loading direction (Mangas Araujo and Brassart, 2025). At this point, the simulation stopped. The numerical procedure is illustrated in Fig. 1(b).

### 2.4 Elastic modulus

Assuming that all chains in the network are in their Gaussian regime in the reference configuration, the shear modulus is given by Gusev (2019) and Alamé and Brassart (2020):

(9) $$G_{0}=\nu_{0}k_{B}T\langle \frac{r_{0}^{2}}{Nb^{2}}\rangle =\nu_{0}k_{B}T{\overset{¯}{\lambda }}_{0}^{2},$$

where $\nu_{0}$ is the initial density of elastically effective chains (number of chains per unit volume in the reference configuration) and ${\overset{¯}{\lambda }}_{0}$ is the average chain pre-stretch: ${\overset{¯}{\lambda }}_{0}\equiv \sqrt{\langle \lambda_{0}^{2}\rangle }$. The notation 〈⋅〉 represents the ensemble average over all the (elastically-effective) chains in the network:

(10) $$\langle \cdot \rangle =\frac{1}{n}\overset{n}{\underset{j=1}{\sum }}\left(\cdot \right).$$

Eq. (9) is exact provided that the network is isotropic, and its validity was verified in our previous works (Alamé and Brassart, 2020, Mangas Araujo et al., 2024). Eq. (9) only recovers the classical expression $G_{0}=\nu_{0}k_{B}T$ in the particular case where ${\overset{¯}{\lambda }}_{0}=1$. For an incompressible, isotropic elastic material, the shear modulus is related to the Young modulus by $E_{0}=3G_{0}$.

## 3 Monodisperse networks

### 3.1 DN simulation results

We first consider monodisperse networks with three different chain lengths N=100,150, and 200 but the same chain density $\nu_{0}b^{3}=1\times 10^{-3}$. In experiments, independent control of chain length and chain density can be achieved in tetra-PEG hydrogels made by crosslinking four-arm macromolecules with well-defined arm length in solution (Sakai et al., 2008). In our simulation, the rms average end-to-end distance is set to ${\overset{¯}{r}}_{0}\equiv \sqrt{\langle r_{0}^{2}\rangle }\approx \sqrt{N}b$ (i.e. ${\overset{¯}{\lambda }}_{0}\approx 1$), so that networks with different N have the same elastic modulus according to Eq. (9), and the chain length only impacts the failure response. For these values of chain density and average pre-stretch, networks with N=100 have an rms average end-to-end distance ${\overset{¯}{r}}_{0}$ close to the end-to-end distance in a diamond lattice, given by $r^{*}=\frac{\sqrt{3}}{4^{1/3}}{\left(\nu_{0}\right)}^{-1/3}$, whereas networks with longer chains are initially interpenetrated (${\overset{¯}{r}}_{0}>r^{*}$). The Kuhn length was fixed at b=1nm, representative of PEG chains (Dittmore et al., 2011), and the temperature was set as T=298K. A critical chain scission threshold η=0.8 was used for all chains. This value is sufficiently high for the chains to reach their strain-stiffening behaviour before breaking while achieving computational efficiency. The effect of the specific value of η on the failure response is discussed below.

**Fig. 2:**
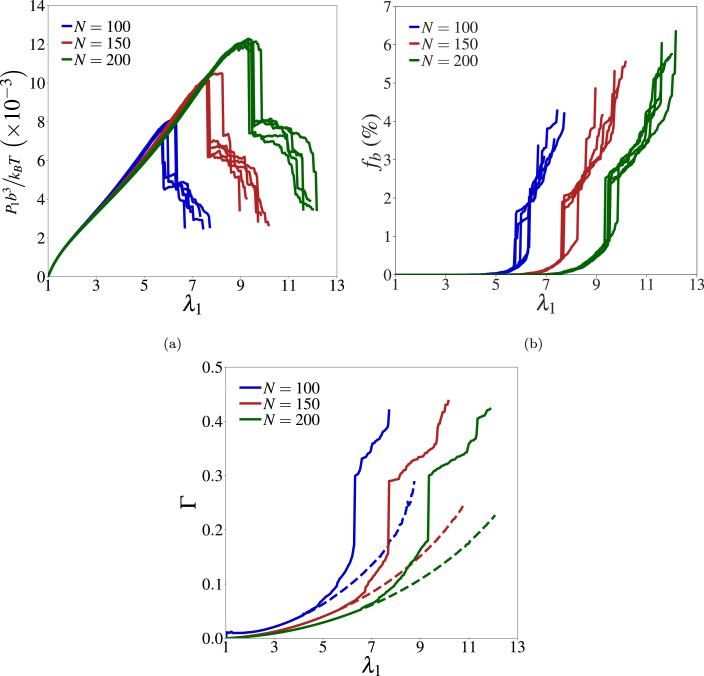
(a) Uniaxial tension response of DNs with $\nu_{0}b^{3}=10^{-3}$, average pre-stretch ${\overset{¯}{\lambda }}_{0}\approx 1$ and chain scission threshold η=0.8 but different chain lengths N. (b) Corresponding evolution of the fraction of broken chains $f_{b}$ during deformation. For each value of N, each curve in the figure corresponds to one network realisation. (c) Evolution of the affinity deviation parameter in one representative network realisation for each value of N (continuous lines). Dashed lines correspond to the same DNs in the nearly-elastic case with η=0.99.

Fig. 2(a) shows stress–stretch curves of monodisperse networks with different chain lengths. The evolution of the fraction of broken chains $f_{b}$ is shown in Fig. 2(b). In these plots, each curve corresponds to one network realisation. The initial overlap of the stress–stretch curves confirms that the networks have the same initial modulus. All curves exhibit a sharp stress drop, coinciding with a sudden increase in the fraction of broken chains, occurring well before the loss of connectivity. Similar results were previously reported by Lei et al. (2025). In experiments, reaching the peak stress usually signals the onset of strain localisation, as a precursor to mechanical failure. We therefore regard the peak stress as the main indicator of the loss of load-bearing capacity. Networks with longer chains show higher peak stretches and ultimate stretches at loss of connectivity. This is because chains can reach a larger stretch $\lambda_{c}=r/r_{0}$ when N increases at fixed η, with maximum chain stretch $\lambda_{c,max}\approx \eta \sqrt{N}$ for $r_{0}\approx \sqrt{N}b$. The maximum stress also increases with N, which can be understood considering that $P_{1}^{e}=\nu \langle \frac{\partial w}{\partial \lambda_{1}}\rangle =\nu \langle fr_{0}\frac{\partial \lambda_{c}}{\partial \lambda_{1}}\rangle$, with ν the current density of elastically-effective chains, showing that the stress depends on the initial end-to-end distance, which increases with N. It should also be noted that the total stress $P_{1}$ is not zero at the loss of connectivity. This is due to remaining chain paths connecting adjacent faces of the simulation volume.

Fig. 2(b) shows that the peak in the stress–stretch curve, indicative of macroscopic failure, is attained after breaking less than 1% of the chains. This is consistent with the small fraction of broken chains reported by Slootman et al. (2020) during the propagation of a crack in an elastomer. These authors used mechanophores to quantify the fraction of broken chains in the fracture process zone, and reported a fraction of broken chains of the order of 0.1% at a distance of 50μm from the crack surface at 25 °C, decreasing approximately exponentially with the distance. Loss of connectivity also occurs at a low fraction of broken chains ($f_{b}\approx$ 6.5% for N=200 and η=0.8). The fraction of broken chains required for the network to lose its connectivity is well below mean-field estimates from percolation theory ($f_{b}\approx 61\text{\%}$ for a diamond lattice (Stauffer and Aharony, 2018)). In classical percolation theory, chains are removed at random. In contrast, in our DN simulations, chains are removed when their force exceeds a critical threshold. The large difference between percolation theory and our numerical simulation results suggests that highly-loaded chains are also those that are most critical to maintain the connectivity of the network.

Fig. 2(c) represents the deviation from affine deformation, defined as (Alamé and Brassart, 2020):

(11) $$\Gamma =\frac{\langle \lambda_{c}-\lambda_{\mathrm{aff}}\rangle }{\lambda_{1}},$$

where $\lambda_{c}$ is the chain stretch in the equilibrated network and $\lambda_{\mathrm{aff}}$ is the stretch of a chain with the same orientation, assuming affine deformation. The figure also shows the affinity deviation parameter in elastic simulations of the same DNs (dashed lines) (In practice, we used η=0.99 in “elastic” simulations to allow highly stretched chains to break and avoid numerical issues associated with chain locking occurring when r/Nb→1). At small macroscopic stretches, the affinity deviation parameter is close to zero, indicating that chains deform mainly affinely. This is consistent with the theoretical result that Gaussian chains deform affinely (Gusev, 2019, Alamé and Brassart, 2020, Mangas Araujo et al., 2024). As the macroscopic stretch increases, non-affine effects become significant. Furthermore, damage by chain scission amplifies the deviation from affine deformation, as shown by the rapid increase of Γ upon approaching the stress drop, compared to simulations in the elastic case.

**Fig. 3:**
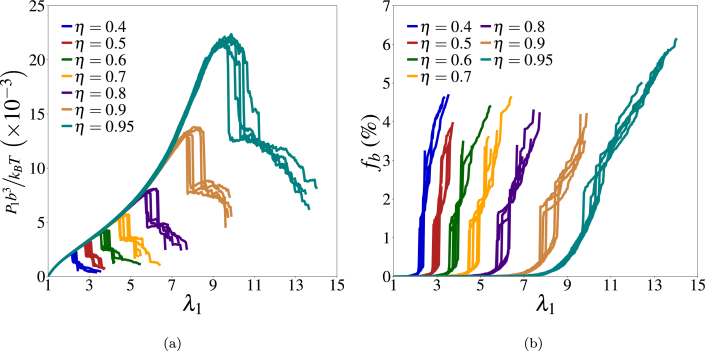
(a) Uniaxial tension response of DNs with $\nu_{0}b^{3}=10^{-3}$, average pre-stretch ${\overset{¯}{\lambda }}_{0}\approx 1$ and chain length N=100, but different chain scission thresholds η. (b) Corresponding evolution of the fraction of broken chains $f_{b}$ during deformation. For each value of η, each curve in the figure corresponds to one network realisation.

The effect of different values of η on the uniaxial tension response is shown in Fig. 3 in the case N=100. Other parameters were kept unchanged. Stress–stretch curves for different values of η show similar trends, involving a sharp stress drop at a low fraction of broken chains, and loss of connectivity reached after breaking only a few percents of chains. This suggests that the specific value of the scission threshold does not affect the damage mechanism at the network scale. However, it does affect the values of the peak stretch and peak stress. This is expected, since chains can stretch further with increasing η, thus sustaining larger forces prior to scission. Interestingly, the fraction of broken chains at the peak and at loss of connectivity remains nearly constant in the range η=0.4 to 0.9, but increases for η=0.95. This likely stems from the highly non-linear response of the FJC model as the end-to-end distance approaches the contour length.

**Fig. 4:**
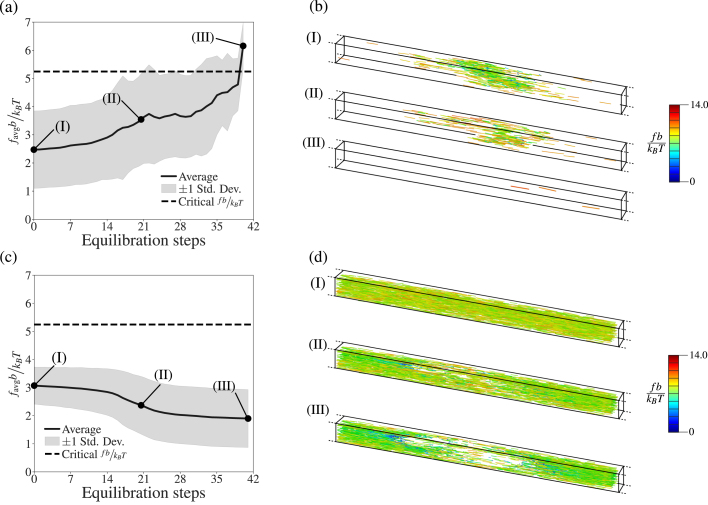
Evolution of the average normalised chain force $\frac{f_{\mathrm{avg}}b}{k_{B}T}$ and snapshots of the chains that break during the drop ((a) and (b)) and top-loaded chains at the onset of the drop ((c) and (d)). From top to bottom, the snapshots show the chains of the corresponding population at the beginning (I), midpoint (II), and end of the sharp stress drop (III). Chains are coloured by their chain force in normalised units. Dashed lines indicate the edges of the simulation box slice shown for visualisation; the full simulation box extends beyond this region.

To understand the micro-mechanisms responsible for the sharp stress drop in Fig. 2(a), we examine the evolution of chain forces during the drop in a representative DN with N=100 and η=0.8. We focus on two populations of chains: (i) chains that eventually break during the drop, and (ii) chains in the top 10% of the most loaded chains at the onset of the drop. We note that the set of chains that eventually break are not necessarily a subset of the 10% most loaded ones. Fig. 4(a) and (c) show the evolution of the average normalised chain force $\frac{f_{\mathrm{avg}}b}{k_{B}T}$ for these two populations of chains as a function of the number of minimisation steps during the first time step of the stress drop. Shaded areas represent one standard deviation relative to the mean. The dashed line marks the critical scission threshold, which corresponds to a normalised chain force of approximately 5.2 according to Eq. (2), evaluated at $\frac{r}{Nb}=\eta =0.8$. Interestingly, Fig. 4(a) shows that the breaking chains are initially below the critical force on average, but that the average force in these chains rises steadily as the drop progresses, ultimately reaching the scission threshold. This suggests that scissions among some of the most loaded chains trigger force redistribution, driving some of them to failure in a cascade. This process can be visualised in Fig. 4(b), which shows snapshots of the breaking chains coloured by their normalised force at three stages of the drop: the beginning, midpoint, and end (from top to bottom). These snapshots confirm that scission-induced force redistribution drives subcritical chains to overstretch and break. In contrast, Fig. 4(c) reveals that the average force among the initially top-loaded chains decreases during the drop, indicating that these chain unload during the stress drop. Corresponding snapshots are shown in Fig. 4(d). The process of force redistribution during the stress drop is further illustrated in a 2D case in Appendix A.

Overall, these results demonstrate that the sharp stress drops seen in Fig. 2(a) result from the failure of a relatively small fraction of chains in a localised region of the network, rather than the uniform overstretching across all chains in the network. We attribute this phenomenon to the existence of an initial distribution in the chain end-to-end distances $r_{0}$, and thus to the initial distribution of chain stretches and forces, as illustrated in Fig. 1(a) . These heterogeneities are controlled by the condition of mechanical equilibrium for a given network topology and are intrinsic to random discrete networks. This makes discrete networks susceptible to localised damage in a random location of the network, even when the network is monodisperse and free from topological defects.

### 3.2 Three-chain and eight-chain models

In addition to providing insights into failure mechanisms, DN simulations are also useful for assessing the predictive capability of micromechanical constitutive theories based on the same single chain model. In Mangas Araujo et al. (2024), we have systematically compared continuum models to DN simulations in the purely elastic case. In this work, we extend our analysis to include damage by chain scission, using the same scission threshold in the DN and continuum theories. We first consider the three-chain (Wang and Guth, 1952) and eight-chain (Arruda and Boyce, 1993) models, which provide an explicit relation between the chain stretch and the macroscopic deformation. As such, they have been widely used to predict the onset of damage in continuum theories (Mao et al., 2017, Talamini et al., 2018, Li and Bouklas, 2020, Arunachala et al., 2023, Mousavi et al., 2025). The three-chain and eight-chain models also constitute upper and lower bounds for the affine full-network model (Wu and Van Der Giessen, 1993), considered in the next section.

In the three-chain model, the elastic energy of the network is estimated by considering three representative chains aligned with the principal stretch directions and deforming according to the macroscopic principal stretches:

(12) $$\psi_{3ch}^{e}=\frac{\nu_{0}}{3}\overset{3}{\underset{j=1}{\sum }}w\left(r_{j}\right),$$

where $r_{j}=\lambda_{j}r_{0}$ (no sum on j). Here we set $r_{0}={\overset{¯}{\lambda }}_{0}\sqrt{N}b$ with ${\overset{¯}{\lambda }}_{0}$ the average chain pre-stretch in the DN, ensuring that the three-chain model and DN model have the same elastic modulus. We extend the three-chain model by assuming that network failure occurs when the chain aligned in the first principal direction reaches its maximum stretch, giving the macroscopic failure stretch as:

(13) $$\lambda_{\mathrm{f,3ch}}=\frac{\eta Nb}{r_{0}}=\frac{\eta \sqrt{N}}{\overset{¯}{\lambda_{0}}},$$

where here ${\overset{¯}{\lambda }}_{0}\approx 1$. In the case where η=1 and ${\overset{¯}{\lambda }}_{0}=1$, the model recovers the classical estimate for the extensibility limit of a single polymer strand, $\sqrt{N}$. The eight-chain model considers a set of eight representative chains aligned with the diagonals of a cube deforming with the principal stretches:

(14) $$\psi_{8ch}^{e}=\nu_{0}w\left(r\right),$$

where $r=\Lambda r_{0}$ and $\Lambda =\sqrt{\frac{\lambda_{1}^{2}+\lambda_{2}^{2}+\lambda_{3}^{2}}{3}}$. The macroscopic failure stretch is obtained by solving:

(15) $$\lambda_{f,8ch}^{2}+\frac{2}{\lambda_{f,8ch}}=\frac{3\eta^{2}N}{{\overset{¯}{\lambda }}_{0}^{2}},$$

for $\lambda_{f,8ch}$.

**Fig. 5:**
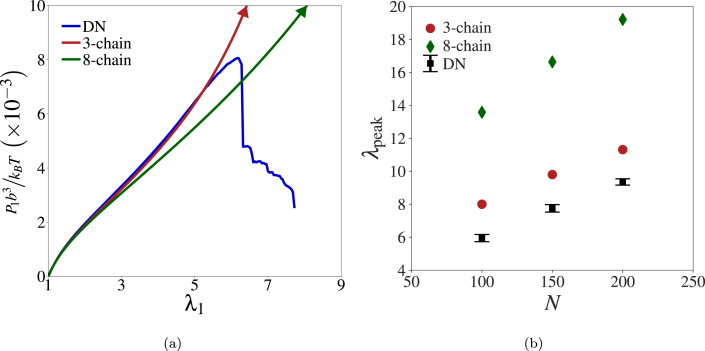
(a) Uniaxial tension response for N=100 and η=0.8, as predicted by the DN model (only one realisation is shown) and the three-chain and eight-chain models. Triangular markers indicate that failure is reached beyond the y-axis limit in the continuum models. (b) Peak stretches $\lambda_{\mathrm{peak}}$ predicted by DN simulations and the three-chain and eight-chain models. DN values represent averages over five realisations, with error bars indicating one standard deviation.

Fig. 5(a) compares DN and analytical model predictions for N=100. The three-chain model accurately captures the stress–stretch response almost up to the peak, whereas the eight-chain model underestimates the stress at moderate deformations. However, both models significantly overestimate $\lambda_{\mathrm{peak}}$ (Fig. 5(b)), with relative errors around 30% and 130% for the three-chain and eight-chain models, respectively. The inability of the three-chain model to predict the onset of failure in the DN is explained by the fact that it considers only three representative chains with uniform initial end-to-end distance and subjected to the affine stretch. In contrast, the DN possesses a distribution of chain initial end-to-end distances. Furthermore, chains in the DN can experience a stretch larger than the affine stretch, as shown in our previous work (Mangas Araujo et al., 2024). By overlooking the highly stretched chains that break first, the three-chain model overestimates the macroscopic stretch at the onset of damage. Nevertheless, the three-chain model provides a good estimate of the DN stress–stretch curve during the elastic part of the response. This means that an accurate prediction of the macroscopic stress–stretch response does not translate into an accurate prediction of damage initiation. The eight-chain model also neglects the heterogeneity in initial end-to-end distance, but assumes that all chains see the same stretch $\Lambda <\lambda_{1}$, leading to a softer response. As a result, the eight-chain model underestimates the undamaged stress–stretch response and leads to an even larger overestimation of the peak stretch. Thus, the eight-chain model provides a poor estimate of the chain stretch for damage prediction. By construction, neither of these models can simulate damage accumulation beyond damage initiation.

### 3.3 Affine and non-affine microsphere models

Results of the previous section highlighted several shortcomings of the simple three-chain and eight-chain models, namely: (1) the consideration of a very small number of representative chains, preventing the simulation of damage accumulation, (2) the consideration of a single initial chain end-to-end distance, overlooking pre-existing heterogeneities in chain end-to-end distances present in the DN, and (3) the use of the affine assumption to calculate the chain stretches, whereas DN chains show clear deviation from affinity. In this section, we address these limitations by considering extended affine and non-affine microsphere theories, building on models from the literature considering damage (Mulderrig et al., 2021) and a distribution of chain pre-stretch (Mangas Araujo et al., 2024). Here, we use the term “extended” to indicate that the microsphere models account for an initial distribution of chain end-to-end distances.

We propose a free energy of the following form:

(16) $$\psi^{e}=\nu_{0}\int_{r_{0}}p_{r_{0}}\left(r_{0}\right)\iint_{S_{u}}H\left(t;r\left(\tau \right)\right)w\left(r\right)dS_{u}dr_{0},$$

where $p_{r_{0}}\left(r_{0}\right)$ represents the distribution of initial end-to-end distances. The notation $\iint_{S_{u}}\left(\cdot \right)dS_{u}$ represents the integration over the unit sphere $S_{u}$:

(17) $$\iint_{S_{u}}\left(\cdot \right)dS_{u}=\frac{1}{4\pi }\int_{0}^{2\pi }\int_{0}^{\pi }\left(\cdot \right)\;\sin\left(\phi \right)\;d\phi d\theta ,$$

where θ and ϕ are the azimuthal and polar angles, respectively. In extended microsphere models, the distribution of chain end-to-end distances $p_{r_{0}}\left(r_{0}\right)$ is taken directly from the DN. In the non-extended models, the distribution reduces to $p_{r_{0}}=\delta ({\overset{¯}{\lambda }}_{0}\sqrt{N}b$) with ${\overset{¯}{\lambda }}_{0}$ the average chain pre-stretch in the DN. Chain scission is introduced in the model through the history variable (Miehe et al., 2010):

(18) $$H\left(t,r\left(\tau \right)\right)=H\left(\eta -\underset{\tau \leq t}{\max}\frac{r\left(\tau \right)}{Nb}\right),$$

where H(x) denotes the Heaviside step function. This variable tracks the maximum normalised elongation $\frac{r}{Nb}$ experienced by a chain up to time t. If this elongation ever exceeds the scission threshold η, H(t) is set to 0, indicating that the chain has broken and can no longer carry load. Otherwise, H(t)=1, and the chain remains intact. We also introduce the macroscopic damage variable D, which serves as a continuum analogue to the fraction of broken chains $f_{b}$ in the DN model and is defined as (Guo and Zaïri, 2021, Mulderrig et al., 2021, Arunachala et al., 2023):

(19) $$D=\int_{r_{0}}p_{r_{0}}\left(r_{0}\right)\iint_{S_{u}}1-H\left(t,r\left(\tau \right)\right)dS_{u}dr_{0}.$$

We developed both affine and fully-relaxed variations of this model. In the affine model, the current end-to-end distance of each chain is given by $r=\lambda_{c}r_{0}$ with $\lambda_{c}=\|Fn_{0}\|$, where $n_{0}$ is the chain orientation in the reference configuration. In the non-affine, or fully-relaxed model, the distribution of chain end-to-end distances is obtained by minimising the free energy of the network under a kinematic constraint. Details of the derivation and numerical implementation of the fully-relaxed model are presented in Appendix B.

**Fig. 6:**
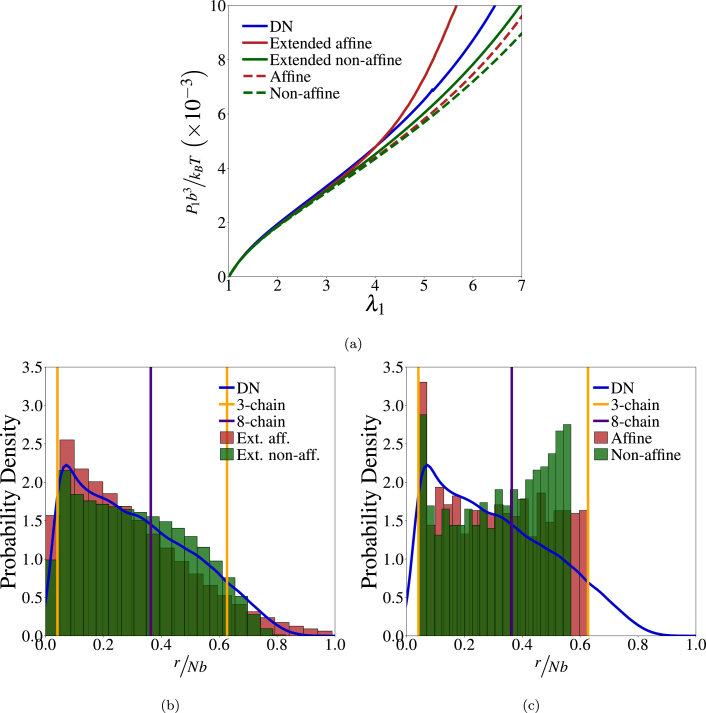
(a) Uniaxial tension response for N=100 in the elastic case, as predicted by the DN model (only one realisation is shown) and extended and non-extended microsphere models. (b)–(c) Distribution of normalised chain end-to-end distance r/Nb at a macroscopic stretch $\lambda_{1}=6.16$ predicted by (b) the extended affine and non-affine microsphere models, and (c) the non-extended affine and non-affine microsphere models, compared to DN simulation results. Normalised chain end-to-end distances predicted by the 3-chain and 8-chain models are shown as vertical lines.

Fig. 6(a) illustrates the performance of microsphere models against DN simulation results for N=100 and in the absence of damage. At small to moderate stretches ($\lambda_{1}≲3$), all models are in good agreement with the DN model. However, the extended affine model is too stiff at large deformations, whereas the extended non-affine model provides a better prediction, although it underestimates the DN response. Non-extended affine and non-affine models both underestimate the DN response, with the affine model always stiffer than the fully-relaxed one. The relative performance of the elastic extended microsphere models can be understood based on their predicted distribution of chain end-to-end distances, shown in Fig. 6(b) at the macroscopic stretch $\lambda_{1}=6.16$, corresponding to the peak stretch in the same DN with damage (η=0.8). Failure stretches predicted by the three-chain and eight-chain models are also shown as vertical lines. Overall, both extended microsphere models well capture the shape of the distribution. The extended affine model overestimates the DN probability density at low and high end-to-end distances, while it underestimates the probability density at intermediate values. The extended non-affine model predicts the opposite trends. By relying on the DN distribution of initial chain end-to-end distances, both extended microsphere models account for chains with end-to-end distances larger than predicted by the three-chain model. The stiffer response of the affine model, compared to the fully-relaxed one and the DN, is attributed to the higher fraction of highly-extended chains in this model, combined with the highly non-linear response of the FJC model as chains approach their contour length. The distribution of chain end-to-end distances in the non-extended microsphere models is shown in Fig. 6(c) for comparison. These models do not capture the DN probability density distribution well, because they assume a uniform initial chain end-to-end distance distribution. Predicted chain end-to-end distances are bounded by the three-chain estimates, with the fully-relaxed model predicting smaller end-to-end distances than the affine one.

**Fig. 7:**
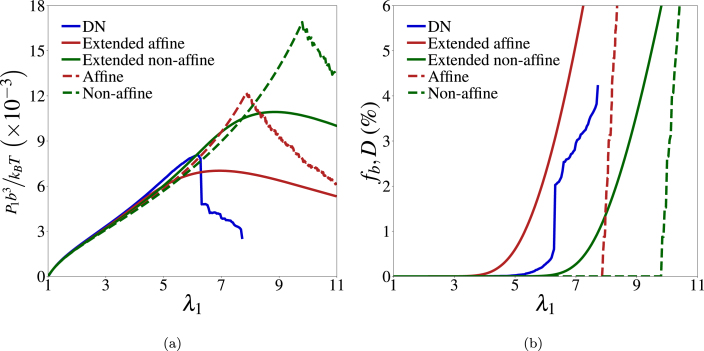
(a) Uniaxial tension response for N=100 and η=0.8, as predicted by the DN model (only one realisation is shown) and microsphere models. (b) Fraction of broken chains $f_{b}$ (DN model) and macroscopic damage variable D (microsphere models) as a function of the macroscopic stretch.

Fig. 7 compares the predictions of affine and non-affine microsphere models to the response of the same DN in the presence of damage with η=0.8. Non-extended models exhibit a sharp peak in the stress–strain response somewhat similar to the DN, however they overestimate the peak stretch (Fig. 7(a)). In the non-extended affine model, the peak coincides with the scission of the chains aligned in the loading direction and coincides with the onset of damage in the three-chain model. The non-extended non-affine model further overestimates the peak stretch because the chain stretch calculated from the fully-relaxed assumption is lower than the affine stretch in the loading direction, as shown in Fig. 6(c). Microsphere models can also predict damage accumulation by subsequently breaking chains in the other directions (Fig. 7(b)). In contrast, extended microsphere models predict a smooth peak and damage accumulation, as a result of the additional sampling over the initial chain end-to-end distances. In addition, the onset of damage and peak stress no longer happen at the same macroscopic stretch. In the affine case, the onset of damage is predicted before the DN peak stretch due to the consideration of highly pre-stretched chains subjected to the affine stretch (Fig. 6(b)), leading to earlier softening and peak stress value close to that of the DN. In the non-affine case, the onset of damage is delayed due to lower predicted chain stretch, leading to an overestimation of the DN peak stretch and peak stress. Relative errors on the peak stretch and stress predicted by the various models and using average DN values as reference are reported in Table 1.

**Table 1:** Relative errors on the peak stretch ($e_{\lambda_{\mathrm{peak}}}$) and peak stress ($e_{P_{\mathrm{peak}}}$) in the different microsphere models, using the average value over five DN realisations as reference.

Model	$e_{\lambda_{\mathrm{peak}}}$ (%)	$e_{P_{\mathrm{peak}}}$ (%)
Extended affine	16.1	10.5
Extended non-affine	48.7	39.3
Affine	32.2	55.4
Non-affine	64.9	115.4

## 4 Bimodal networks

We next consider the deformation and failure response of networks with a bimodal distribution of chain parameters. Two representative cases are successively considered: (i) chains with two different lengths but same strength, and (ii) chains with the same length, but different scission thresholds. All DN results presented in this section were obtained taking b=1nm, $\nu_{0}b^{3}=1\times 10^{-3}$, and T=298 K.

### 4.1 Bimodal chain length distribution

We first consider networks with bimodal chain length but same chain density. This scenario is inspired by the bimodal tetra-PEG hydrogels considered by Kondo et al. (2013). Networks with a bimodal chain length distribution were generated from a base network with ${\overset{¯}{r}}_{0}\approx 10b$ (close to the end-to-end distance in a diamond lattice at this chain density) by randomly assigning each chain a number of Kuhn segments $N_{S}=100$ or $N_{L}=200$, according to prescribed fractions of short and long chains $\varphi_{S}$ and $\varphi_{L}$ with $\varphi_{S}+\varphi_{L}=1$. Networks were then equilibrated to obtain the reference configuration. Under these conditions, we found that the rms average end-to-end distance in the reference configuration ${\overset{¯}{r}}_{0}$ remained nearly constant across the different fractions of short and long chains. This means that the average pre-stretch changed with $\varphi_{L}$, ranging from ${\overset{¯}{\lambda }}_{0}\approx 1$ for $\varphi_{L}=0$ to ${\overset{¯}{\lambda }}_{0}\approx 0.7$ for $\varphi_{L}=1$. All chains were given the same scission threshold η=0.8, and thus the same critical force for chain scission (“chain strength”), given that the chain force only depends on the ratio r/Nb according to the FJC model (for a fixed Kuhn length and fixed temperature). For the bimodal networks, five realisations were generated by randomly assigning the chain lengths in a given base topology. For the monodisperse networks with $\varphi_{L}=0$ and $\varphi_{L}=1$, five realisations of random networks were produced.

**Fig. 8:**
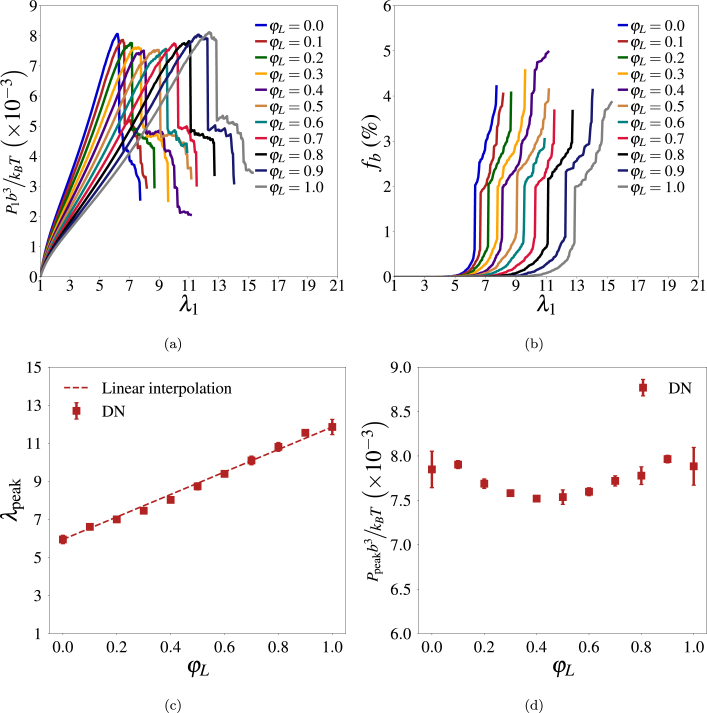
(a) Uniaxial tension response of DNs with different fractions of long chains $\varphi_{L}$. (b) Evolution of the fraction of broken chains $f_{b}$. In (a) and (b), only one representative curve per condition is shown for clarity. (c) Peak stretch $\lambda_{\mathrm{peak}}$, and (d) peak nominal stress $P_{\mathrm{peak}}$ as a function of the fraction of long chains $\varphi_{L}$.

Fig. 8(a) shows stress–stretch curves of discrete networks with bimodal chain length for selected values of $\varphi_{L}$. As the fraction of long chains increases, the stiffness decreases due to the reduction in average chain pre-stretch. In addition, the peak stress remains roughly constant while the stretchability increases, leading to an increase in toughness (here defined as the area under the stress–stretch curve). Similar to the monodisperse case, a sharp stress drop is observed for all fractions of long chains, corresponding to a sharp increase in the fraction of broken chains, shown in Fig. 8(b). Interestingly, the fraction of broken chains required for loss of connectivity seems roughly independent of the fraction of long chains, although a significant statistical variation is seen across repeats.

The DN peak stretch $\lambda_{\mathrm{peak}}$ is represented as a function of the fraction of long chains $\varphi_{L}$ in Fig. 8(c), showing an approximately linear dependence. The dashed line represents a linear interpolation between peak stretch values of the network with only short and long chains, respectively. It corresponds to the 3-chain estimate for a monodisperse network with average chain length $N_{\text{avg}}=\varphi_{S}N_{s}+\varphi_{L}N_{L}$:

(20) $$\lambda_{f}=\alpha \frac{\eta N_{\text{avg}}b}{{\overset{¯}{r}}_{0}},$$

where the factor α corrects for the overestimation of the peak stretch by the three-chain model already noted in Section 3.2. Also remember that ${\overset{¯}{r}}_{0}$ is independent of $\varphi_{L}$ in our simulations. The linear scaling (20) is consistent with the experimental results of Kondo et al. (2013) for the rupture stretch of tetra-PEG gels with bimodal chain length. These authors proposed a similar relation, assuming that the initial average end-to-end distance only depends on chain density. An (approximately) linear increase in rupture stretch with the fraction of short chains was also reported in model bimodal PDMS elastomers by Andrady et al., 1980b, Andrady et al., 1980a. However, these elastomers likely contained a large number of trapped entanglements as well as an inhomogeneous distribution of the chains, making direct comparison challenging (Urayama et al., 1998, Viers and Mark, 2005, Kondo et al., 2013).

The evolution of the peak stress $P_{\mathrm{peak}}$ with the fraction of long chains is shown in Fig. 8(d), showing little variation with $\varphi_{L}$. This is also consistent with the three-chain estimate of the peak stress for a monodisperse network with average chain length $N_{\text{avg}}$, where the maximum stress scales as $P_{f}∼\nu_{0}r_{0}f_{c}$, noting that critical force and the initial end-to-end distance are independent of the chain length in our networks. However, this result contradicts the experimental results of Kondo et al. (2013), who reported a large increase in rupture stress with the fraction of long chains.

**Fig. 9:**
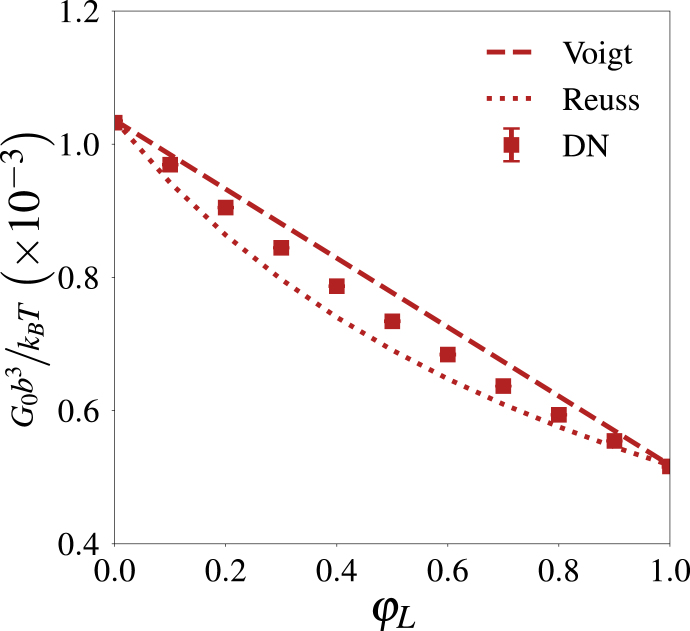
Elastic shear modulus $G_{0}$ of DNs with a bimodal chain length distribution as a function of the fraction of long chains $\varphi_{L}$. DN data points represent the average over five network repeats. Error bars are included but are barely visible. “Voigt” and “Reuss” averages are shown as dashed and dotted lines, respectively.

Fig. 9 shows the dependence of the shear modulus $G_{0}$ on the fraction of long chains $\varphi_{L}$. The figure also shows the predictions given by simple mixture rules, parallelling the classical Voigt and Reuss bounds of composite theory:

(21) $$G_{V}=\varphi_{S}G_{S}+\varphi_{L}G_{L},$$

and:

(22) $$G_{R}={\left(\frac{\varphi_{S}}{G_{S}}+\frac{\varphi_{L}}{G_{L}}\right)}^{-1}.$$

where $G_{S}$ and $G_{L}$ are the shear moduli of monodisperse networks with only short and long chains, respectively. The shear modulus of the bimodal DNs lies within these two estimates. In the context of DNs, the physical significance of these two estimates is the following. The “Voigt” average (21) is obtained from the exact relation (9) by assuming that short and long chains have the same square average initial chain end-to-end distance: ${\langle r_{0}^{2}\rangle }_{S}={\langle r_{0}^{2}\rangle }_{L}=\langle r_{0}^{2}\rangle$, where ${\langle \cdot \rangle }_{S}$ and ${\langle \cdot \rangle }_{L}$ represent ensemble averages over the populations of short and long chains, respectively. Eq. (21) follows, considering that $\langle r_{0}^{2}\rangle$ was found to be largely insensitive to $\varphi_{L}$ in these networks. The “Reuss” average is obtained from Eq. (9) by treating the network as a monodisperse network with average chain length $N_{\text{avg}}$ and also assuming that $\langle r_{0}^{2}\rangle$ is independent of $\varphi_{L}$.

**Fig. 10:**
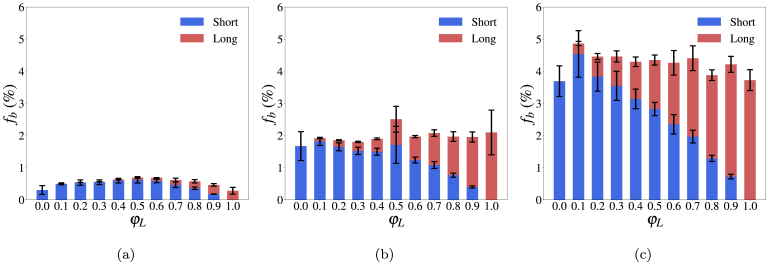
Contributions of short and long chains to the total fraction of broken chains $f_{b}$ for different values of $\varphi_{L}$ at (a) the peak, (b) the end of the stress drop, and (c) loss of connectivity. Bar heights represent the average over five network realisations and error bars indicate one standard deviation.

Fig. 10 shows the relative contributions of short and long chains to the total fraction of broken chains $f_{b}$ for different values of $\varphi_{L}$. The contribution of short and long chains to the total fraction of broken chains $f_{b}$ varies with the stage of deformation and the fraction of long chains $\varphi_{L}$. Three key deformation stages are considered: (a) the peak, (b) the end of the stress drop, and (c) loss of connectivity. At the peak of the stress–stretch curve (Fig. 10(a)), scissions occur predominantly among the short chains except for $\varphi_{L}=0.9$ and $\varphi_{L}=1$. However, the relative fraction of long chains increases with further deformations (Fig. 10(b)–(c)). In particular, the relative fraction of long chains to the overall fraction of broken chains is approximately $\varphi_{L}$ at the loss of connectivity. Fig. 10(c) also confirms that the overall fraction of broken chains $f_{b}$ is roughly constant across the values of $\varphi_{L}$.

**Fig. 11:**
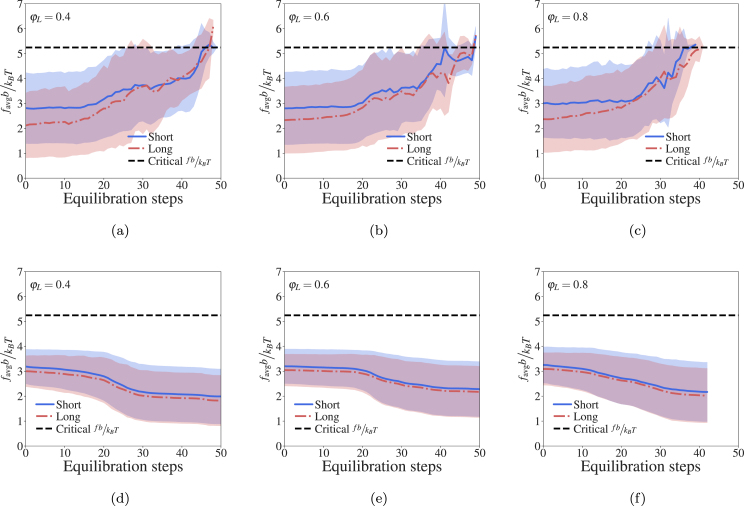
Evolution of the normalised average force $\frac{f_{\mathrm{avg}}b}{k_{B}T}$ per chain type during the stress drop in bimodal DNs with varying fractions of long chains $\varphi_{L}=0.4$ ((a), (d)), 0.6 ((b), (e)), and 0.8 ((c), (f)). Figs (a)–(c) show the average force for the chains that eventually break, while (d)–(f) show the average force of the top 10% most loaded chains at the onset of the drop. Short and long chains are plotted separately, with shaded regions representing one standard deviation. The horizontal dashed line marks the scission threshold, the same for short and long chains.

Fig. 11 shows the evolution of the average chain force during the equilibration sub-steps of the stress drop in two populations of chains: (i) those that eventually break during the drop, and (ii) the top 10% most loaded chains at the start of the drop. Fig. 11(a)–(c) show this evolution for the breaking chains in representative DNs with $\varphi_{L}=0.4,0.6$ and 0.8 respectively. Just before the stress drop, the average chain force in short and long chains is lower than the critical force for chain scission, and the average force in the long chains is also significantly smaller than in the short chains for all $\varphi_{L}$. In contrast, the difference in average chain forces in short and long chains in the top 10% most loaded chains is much smaller at the same time point (Fig. 11(d)–(f)). This suggests that failure starts in a region of the network where there is a large difference in chain force between short and long chains. During the stress drop, the redistribution of forces due to chain scission progressively drives more chains towards the critical breaking threshold and the difference in average force between short and long chains reduces, indicating that both chain types contribute to failure. Simultaneously, short and long chains in the top 10% most loaded chains unload. Thus, the failure mechanism is similar to the monodisperse case, with failure resulting from a scission cascade within a spatially localised cluster of near-critical chains, while chains away from the failure process zone unload.

### 4.2 Bimodal chain strength distribution

We next consider networks with a bimodal chain strength, inspired by tetra-PEG gels containing mechanophores with different mechanochemical reactivities, leading to “weak” and “strong” chains (Beech et al., 2023). Networks with a bimodal chain strength were generated from a base network with N=100 and ${\overset{¯}{r}}_{0}\approx \sqrt{N}b$ by randomly assigning each chain a scission threshold $\eta_{w}=0.4$ or $\eta_{s}=0.8$, according to a prescribed fraction of weak and strong chains $\varphi_{w}$ and $\varphi_{s}$ with $\varphi_{w}+\varphi_{s}=1$. These values of η were selected to produce a rupture–force ratio close to the one reported in Beech et al. (2023). For $0<\varphi_{s}<1$, five realisations of each condition were generated by randomly assigning the chain strengths in a given base topology. For $\varphi_{s}=0$ or $\varphi_{s}=1$, five realisations of random networks were used.

**Fig. 12:**
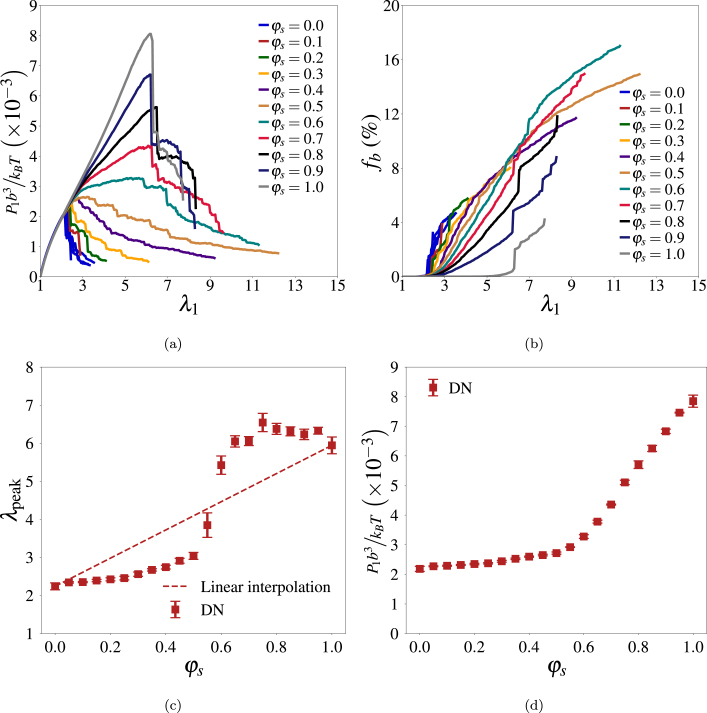
(a) Uniaxial tension response of DNs with different fractions of strong chains $\varphi_{s}$. (b) Evolution of the fraction of broken chains $f_{b}$. In (a) and (b), only one representative curve per condition is shown for clarity. (c) Peak stretch $\lambda_{\mathrm{peak}}$, and (b) peak nominal stress $P_{\mathrm{peak}}$ as a function of the fraction of strong chains $\varphi_{s}$. DN data points represent the average over five network repeats at a given fraction, and error bars correspond to one standard deviation.

Fig. 12(a) shows representative stress–stretch curves of networks with bimodal chain strength for selected values of $\varphi_{s}$. The corresponding evolution of the fraction of broken chains is shown in Fig. 12(b). Compared to a network of weak chains, increasing the fraction of strong chains increases the network strength without impacting the stiffness, as expected from Eq. (9). Interestingly, the sharp stress drop and sharp rise in the fraction of broken chains almost disappear at intermediate values of $\varphi_{s}$ in the approximate range 0.4–0.6. This suggests that the scission cascade triggered by force redistribution is attenuated when weak and strong chains are present in similar proportions. Fig. 12(b) also shows that the critical value of $f_{b}$ at which network connectivity is lost varies considerably with $\varphi_{s}$. As the fraction of strong chains further increases, sharp stress drops reappear, but are now preceded by a softening regime, during which scissions accumulate progressively over a broader stretch range. As $\varphi_{s}$ approaches 1, the stress–stretch response tends to that of a unimodal network of strong chains. Fig. 12(c) shows the dependence of $\lambda_{\mathrm{peak}}$ on $\varphi_{s}$. The peak stretch follows a sigmoid evolution. For $\varphi_{s}≲0.5$, the peak stretch remains nearly constant despite the addition of strong chains. The peak stretch then increases sharply in the range $0.5≲\varphi_{s}≲0.7$. For $\varphi_{s}≳0.7$, the peak stretch approaches and even surpasses the peak stretch of the unimodal network of strong chains. The sigmoid-like evolution of the peak stretch with increasing fractions of strong chains qualitatively resembles that reported in the experiments (Beech et al., 2023). As noted by these authors, the sigmoid evolution of the peak stretch differs from a simple rule of mixture based on an average chain scission threshold: $\eta_{\mathrm{avg}}=\varphi_{w}\eta_{w}+\varphi_{s}\eta_{s}$:

(23) $$\lambda_{f}=\alpha \frac{Nb}{{\overset{¯}{r}}_{0}}\eta_{\mathrm{avg}},$$

where α is a parameter correcting for the inaccuracies of this estimate in the case of a monodisperse network with uniform chain strength. Eq. (23) is represented as a dashed line in Fig. 12(c). The evolution of the peak nominal stress $P_{\mathrm{peak}}$ is shown in Fig. 12(d). Similar to the peak stretch, the peak nominal stress remains nearly unchanged for small to intermediate fractions of strong chains ($\varphi_{s}≲0.5$), before increasing almost linearly with $\varphi_{s}$.

**Fig. 13:**
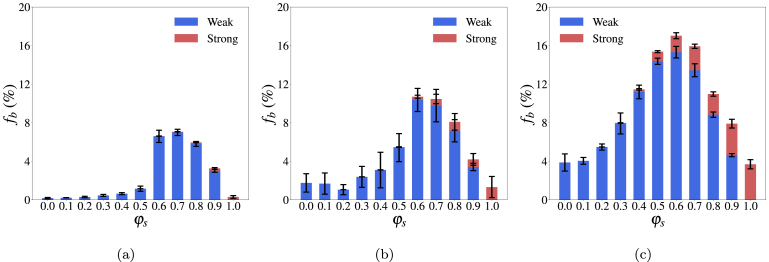
Breakdown of the total fraction of broken chains $f_{b}$ into individual contributions from each chain type for different fractions of strong chains $\varphi_{s}$ at (a) the peak, (b) the end of the largest stress drop, and (c) loss of connectivity. Bars represent the average over five network realisations and error bars indicate one standard deviation.

Fig. 13 shows the relative proportions of strong and weak chains in the total fraction of broken chains $f_{b}$ at three key deformation stages: (a) the peak, (b) the end of the sharp stress drop, and (c) loss of connectivity. For DNs in which no pronounced stress drop is observed, the data are extracted at the end of the largest stress drop. At all deformation stages, chain scission occurs predominantly in weak chains. Strong chains remain essentially intact up to the peak (Fig. 13(a)), with significant scission of strong chains occurring only after the largest stress drop (Fig. 13(b,c)), and in networks where the strong chains outnumber the weak ones ($\varphi_{s}≳0.5$). Fig. 13(c) also confirms that the fraction of broken chains needed to lose connectivity varies non-monotonously with the fraction of strong chains, reaching a maximum at $\varphi_{s}=0.6$.

**Fig. 14:**
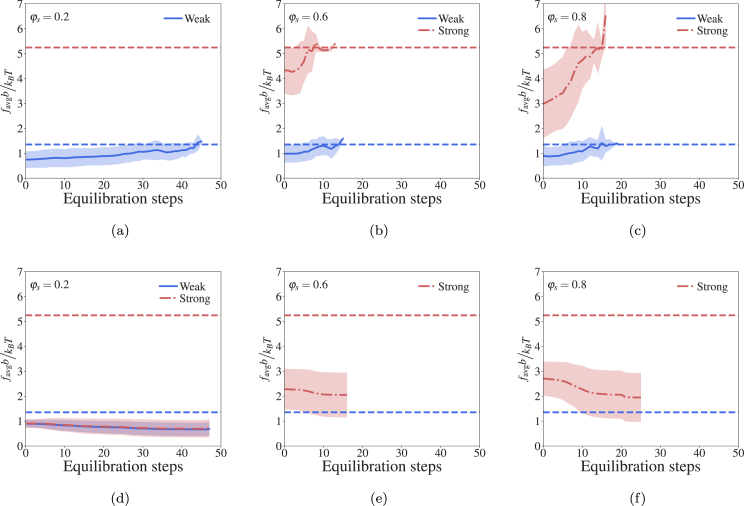
Evolution of the normalised average force $\frac{f_{\mathrm{avg}}b}{k_{B}T}$ per chain type during the largest stress drop for bimodal DNs with varying fractions of strong chains $\varphi_{s}=0.2$ ((a), (d)), 0.6 ((b), (e)), and 0.8 ((c), (f)). Panels (a)–(c) present this evolution for the chains that eventually break, while (d)–(f) show the force redistribution among the top 10% most loaded chains at the onset of the drop. Weak and strong chains are plotted separately, with shaded regions representing one standard deviation. The horizontal dashed line marks the scission threshold for weak and strong chains.

Fig. 14 shows the evolution of the average normalised chain force $\frac{f_{\mathrm{avg}}b}{k_{B}T}$ at the onset of the drop in (a)–(c) the chains that eventually break, and (d)–(f) the top 10% most highly loaded chains, for (a)–(d) $\varphi_{s}=0.2$, (b)–(e) $\varphi_{s}=0.6$ and (c)–(f) $\varphi_{s}=0.8$. The threshold forces for the two populations of chains are shown as dashed lines. For $\varphi_{s}=0.2$, only weak chains break during the stress drop (Fig. 14(a)), while the average force in the 10% most loaded chains is similar in strong and weak chains and decreases during the drop (Fig. 14(d)). For $\varphi_{s}=0.6$ and $\varphi_{s}=0.8$, both weak and strong chains simultaneously approach their respective scission thresholds during the stress drop due to force redistribution triggered by prior scissions (Fig. 14(b)–(c)). In particular, the average force carried by the strong chains is significantly larger than the average force in the weak chains, due to their higher scission threshold. Also, weak chains are absent from the set of the top 10% most loaded chains at these fractions (Fig. 14(e)–(f)), partly due to their lower scission threshold, but also possibly because highly loaded weak chains have already broken by the time the onset of the sharp stress drop is reached.

Based on these results, we introduce three failure regimes, based on the fraction of strong chains (the values of $\varphi_{s}$ delimiting these regimes are only indicative):

• In regime I ($\varphi_{s}≲0.4$), failure is governed by the weak chains, which are both the majority and closer to their scission threshold. This facilitates the formation of localised clusters of critical chains that can trigger a chain scission cascade. The network effectively behaves as a unimodal network of weak chains, with stretch response characterised by a steep stress drop with minimal pre-drop softening. In this regime, the fraction of strong chains has a small impact on the peak stretch.
• In regime II ($0.4≲\varphi_{s}≲0.7$), weak chains remain preferential scission sites, but their population size is comparable to that of strong chains. As a result, weak chains approaching failure are increasingly surrounded by strong chains far from their scission threshold. This disperses the force redistribution and inhibits the formation of clusters of critical chains capable of triggering a scission cascade, and diffuse chain scission events. This translates in an absence of sharp stress drops in the stretch-stress curve and a smoother increase in the fraction of broken chains. Regime II is also characterised by a sharp increase in peak stretch with $\varphi_{s}$.
• In regime III ($0.7≲\varphi_{s}<1$), weak chains are still the first ones to break, but they are now the minority and in insufficient number to cause a scission cascade. The accumulation of diffuse scission events instead leads to pre-stress drop softening in the stretch-stress curve. However, as deformation increases, the strong chains accumulate higher forces and eventually reach their breaking threshold. Once a sufficiently large cluster of near-critical chains is created, a cascade can emerge, producing the delayed but sharp stress drop observed in this regime. In Regime III, the peak stretch fluctuates around a plateau, and is slightly larger than the peak stretch of the unimodal network of strong chains.

## 5 Discussion

### 5.1 Network structure

Our DN simulations were based on highly idealised networks, inspired by tetra-PEG hydrogels. These hydrogels are obtained by the AB coupling of four-arm macromolecular precursors in solution (Sakai et al., 2008). Experimental preparation parameters include the molecular weight $M_{w}$ of the precursors (mass per mol), which is proportional to N, and the volume fraction of polymer $\phi_{0}$, which can be related to the chain density in the crosslinked network by:

(24) $$\nu_{0}=\frac{2\phi_{0}\rho N_{a}}{M_{w}},$$

where ρ is the density of PEG and $N_{a}$ is the Avogadro number. Note that this relation assumes that all the arms of the precursors react. However, quantitative comparisons between DN simulations and experimental results are challenging for several reasons. First, the network of tetra-PEG hydrogels is not perfect, but contains topological defects in the form of unreacted dangling chains and loops (Lange et al., 2011). As shown in our earlier work (Alamé and Brassart, 2020), both dangling ends and loops significantly impact the elastic modulus and extensibility of DNs. While the fraction of dangling ends can be measured and even tuned in experiments (Nishi et al., 2012), the fraction of loops of different types is more difficult to quantify and rarely reported in experimental studies. Second, the actual average end-to-end distance (or pre-stretch) is not precisely known in the experiments. It has been claimed that the four-arm precursors behave as impenetrable spheres in solution, leading to networks with an ideal diamond-like structure (Matsunaga et al., 2009). In this picture, the average chain end-to-end distance would approximately be given by that in a diamond network, ${\overset{¯}{r}}_{0}∼\frac{\sqrt{3}}{4^{1/3}}\nu_{0}^{-1/3}$, independent of the chain length. However, this picture was contradicted by numerical simulations (Schwenke et al., 2011), showing that the classical scaling for the polymer chain end-to-end distance with the number of Kuhn segments holds, and that the four-arm precursors can interpenetrate. Experimental data of Kondo et al. (2013) for the elastic modulus of tetra-PEG gels with bimodal chain length supports the second hypothesis, showing nearly constant elastic modulus for different fractions of short and long chains at the same chain density. However, scaling of the maximum extensibility of tetra-PEG gels with the precursor molecular weight $M_{w}$ reported by Akagi et al. (2013) and Kondo et al. (2013) supports the diamond-like picture. Our DN simulations for monodisperse networks in Section 3.1 correspond to the second hypothesis, giving an elastic modulus independent of N, whereas DN simulations of networks with bimodal chain length in Section 4.1 correspond to the first hypothesis, leading to an elastic modulus decreasing with the fraction of long chains. Finally, and more fundamentally, more recent experimental results by the Sakai group showed that the modulus of tetra-PEG gels is not only governed by entropic elasticity, but also by energetic effects attributed to interactions between the polymer and water (Yoshikawa et al., 2021). Accounting for this effect was shown to be necessary to rationalise the effects of $M_{w}$, $\phi_{0}$ and fraction of dangling ends on the elastic modulus reported in previous experimental studies, as reviewed by Sakumichi et al. (2021). For all these reasons, we did not attempt to quantitatively compare DN simulation results to experimental data for tetra-PEG gels.

More conventional synthesis methods of rubbery networks include free-radical polymerisation of hydrogels (Yang et al., 2019, Kim et al., 2021, Wang et al., 2023b) and vulcanisation of elastomers (Treloar, 1973, Erman and Mark, 1997). Such networks are polydisperse, and the volume fraction of polymer $\phi_{0}$ is coupled to the chain density by:

(25) $$\nu_{0}=\frac{\phi_{0}\rho N_{a}}{\overset{¯}{N}M_{0}},$$

where $M_{0}$ is the molecular weight of the monomer and $\overset{¯}{N}$ is the average number of Kuhn segments in polymer strands between two crosslink points. The key difference with Eq. (24) is $\overset{¯}{N}$ is not known a priori. Inserting Eq. (25) into relation (9) for the elastic modulus, and assuming that polymer strands between crosslink points adopt their random-walk end-to-end distance so that ${\overset{¯}{\lambda }}_{0}=1$, we obtain the familiar formula for the modulus (Rubinstein and Colby, 2003):

(26) $$G_{0}=\frac{\phi_{0}\rho N_{a}k_{B}T}{\overset{¯}{N}M_{0}},$$

which predicts a decrease in modulus as $\overset{¯}{N}$ increases at constant polymer volume fraction, as also confirmed experimentally (Zhou et al., 2020, Kim et al., 2021, Zhong et al., 2024). In contrast, our DN simulations for monodisperse DN with different chain lengths (Fig. 2) predicted a modulus independent of N. As previously explained, this is because the chain length was varied while keeping both the chain density and average pre-stretch constant, implying that the volume fraction of polymer changed with N. Equivalent monodisperse networks more representative of random crosslinking conditions could in principle be obtained within our simulation framework by changing the chain length and chain density simultaneously, at fixed average pre-stretch and fixed polymer volume fraction.

More realistic DN simulations should however consider polydispersity. In our previous work (Mangas Araujo et al., 2024), we have investigated the elastic response of polydisperse networks with a log-normal distribution of the chain length. We showed that the extensibility limit of polydisperse networks decreases as the standard deviation of the distribution increases at constant average number of Kuhn segments per chain. We attributed this effect to the locking of the shortest chains in the distribution as the macroscopic stretch increases. Based on these previous results, we would expect the failure response of polydisperse networks to be dominated by the failure of the short chains, similar to the bimodal chain length case. However, the network generation algorithm for polydisperse networks used in Mangas Araujo et al. (2024) is limited, because it decouples the generation of the topology from the assignment of chain length based on a pre-defined distribution. This contrasts with real networks, where the chain length distribution follows from the random crosslinking process. Future works should focus on developing a network generation algorithm that mimics the gelation process for the system of interest (including for example tetra-PEG gels). Such an approach would naturally couple the chain length, chain density and distribution of initial end-to-end distances, for given preparation conditions. It would also naturally produce topological defects such as dangling ends and loops. Examples of such algorithms can be found in Gusev (2019) and Wagner et al. (2022).

Entanglements constitute another class of network defects that can significantly impact the deformation and failure response (Kim et al., 2021, Zheng et al., 2022). In a first, naive approach, entanglements could be modelled in the DN as additional crosslink points. However, further developments of the DN framework would be needed to account for the sliding and release of entanglements, see for example (Assadi et al., 2025).

### 5.2 Chain behaviour

To simplify the analysis and the comparison with analytical models, we have described polymer chains using the widely-used FJC model, augmented with a scission criterion with a fixed threshold. However, the FJC model is a purely entropic model which neglects energetic contributions due to bond stretching and bond angle opening. Zhu and Brassart (2025) recently showed using a statistical mechanics model that both energetic effects significantly impact the stretching response of a polymer chain at high stretches. In particular, bond stretching and bond angle opening allow the chains to be stretched well beyond their nominal contour length Nb.

The scission threshold η=0.8 used in most of our simulations is also unrealistic in view of real scission forces in polymer chains, which are generally reported in the range 500 pN to a few nN (Beyer and Clausen-Schaumann, 2005). Using b=1nm and T=298 K, the FJC model predicts rupture forces of approximately 21 pN for η=0.8, 82 pN for η=0.95, and 0.4 nN for η=0.99. Reaching realistic chain forces using the FJC model would require a threshold value very close to 1, which introduces numerical challenges in the DN model due to steep gradients during energy minimisation. Nevertheless, our results show that the failure mechanism in the DN is independent of the specific choice of η. Considering more advanced chain models accounting for deformable bonds (Mao et al., 2017, Lavoie et al., 2020, Zhu and Brassart, 2025) would, in principle, address this issue by suppressing the force singularity as r/Nb→1.

Finally, chain scission is a rate-dependent process. In our recent work (Mangas Araujo and Brassart, 2025), we have described rate-dependent chain scission within a DN framework by using an Eyring-type, force-biased kinetic model implemented using a Kinetic Monte Carlo (KMC) algorithm. However, we still used the FJC model to describe the chains, and used the chain force in the kinetic model, instead of the bond force. Future works should combine advanced chain models with a kinetic model of chain scission, providing a more realistic description of the failure process. In particular, it will be important to verify whether the failure mechanisms identified in the present work still hold during rate-dependent failure, and whether the use of a fixed threshold as a chain scission criterion coincides with the limit of fast rate loading, as suggested in the work of Mulderrig et al. (2023).

### 5.3 Perspectives for continuum constitutive theories

Despite the idealised representation of the network structure and chain behaviour, DN models constitute a useful tool to systematically assess the performance of micromechanical constitutive models. This is because DN and constitutive models can be formulated using the same single chain model and the same chain scission criterion, mainly differing in the chain stretch calculation. One key message from this work is that an accurate prediction of the macroscopic stress response in the elastic case without damage does not translate into an accurate prediction of the onset of damage. For example, the three-chain model provides the best estimate of the DN elastic response of all models tested so far, but significantly overestimates the macroscopic stretch at which damage starts (Fig. 5(a)). Among microsphere theories considered here, the most sophisticated extended non-affine model gives the best predictions in elasticity (Fig. 6(a)), but not in the presence of damage where the extended affine model more accurately predicts the peak stretch (Fig. 7(a)). However, both extended microsphere models predict a soft peak very different from the sharp peak observed in DN simulations.

The inability of the extended non-affine microsphere model to capture damage can be explained based on the predicted distribution of chain end-to-end distances during elastic deformations (Fig. 6(b)), in particular the fact that it misses highly-stretched chains, resulting in a delay in the onset of damage compared to the DN. To address this, DN simulations could be used to identify and calibrate an improved localisation rule to better predict the chain stretch distribution during elastic deformations of the network. Ideally, one would also need to develop a model to predict the distribution of chain end-to-end distances in the reference configuration, given its central role in the model performance. The onset and accumulation of damage pose an additional challenge. In the DN, damage propagates by force redistribution in neighbouring chains. This is a local effect which cannot be explicitly described in a continuum constitutive model where only one representative chain stretch is used for each direction and initial end-to-end distance. A better fit between microsphere damage models and DN simulation results could in principle be obtained by introducing an empirical damage function in the microsphere integral, as considered for example by Mulderrig et al. (2021), using DN simulations for fitting. Finally, we note that extended microsphere models can also be developed for polydisperse networks, at the expense of an additional integration over the chain length distribution. Polydisperse microsphere models with and without damage have been developed in several works, e.g. Diani and Le Tallec, 2019, Mulderrig et al., 2021 and Mangas Araujo et al. (2024).

## 6 Conclusions

We have used Discrete Network (DN) simulations to investigate the failure mechanisms in model networks, including monodisperse networks and networks with bimodal distributions of chain length or chain strength. Our results highlight the highly heterogeneous nature of the failure process, involving the scission of a relatively small fraction of chains in an avalanche-like process governed by force redistribution as the chains fail. Localised failure is attributed to the inherent inhomogeneous distribution of chain stretches that necessarily pre-exist in random networks. Simple micromechanical models of rubber elasticity theory fail to predict network failure because they neglect stretch heterogeneities. Extended microsphere models that account for the initial distribution of chain pre-stretch better describe the distribution of chain end-to-end distances during elastic deformation, as well as progressive damage accumulation, but fail to reproduce the sharp stress drop observed in DN simulations, pointing to the need for advanced continuum theories.

## CRediT authorship contribution statement

**Lucas Mangas Araujo:** Writing – original draft, Software, Methodology, Investigation, Formal analysis, Conceptualization. **Laurence Brassart:** Writing – original draft, Writing – review & editing, Supervision, Methodology, Funding acquisition, Formal analysis, Conceptualization.

## Declaration of competing interest

The authors declare the following financial interests/personal relationships which may be considered as potential competing interests: Laurence Brassart reports financial support was provided by UK Research and Innovation. Lucas Mangas Araujo reports was provided by University of Oxford Clarendon Fund. If there are other authors, they declare that they have no known competing financial interests or personal relationships that could have appeared to influence the work reported in this paper.

## Figures and Tables

**Fig. 15:**
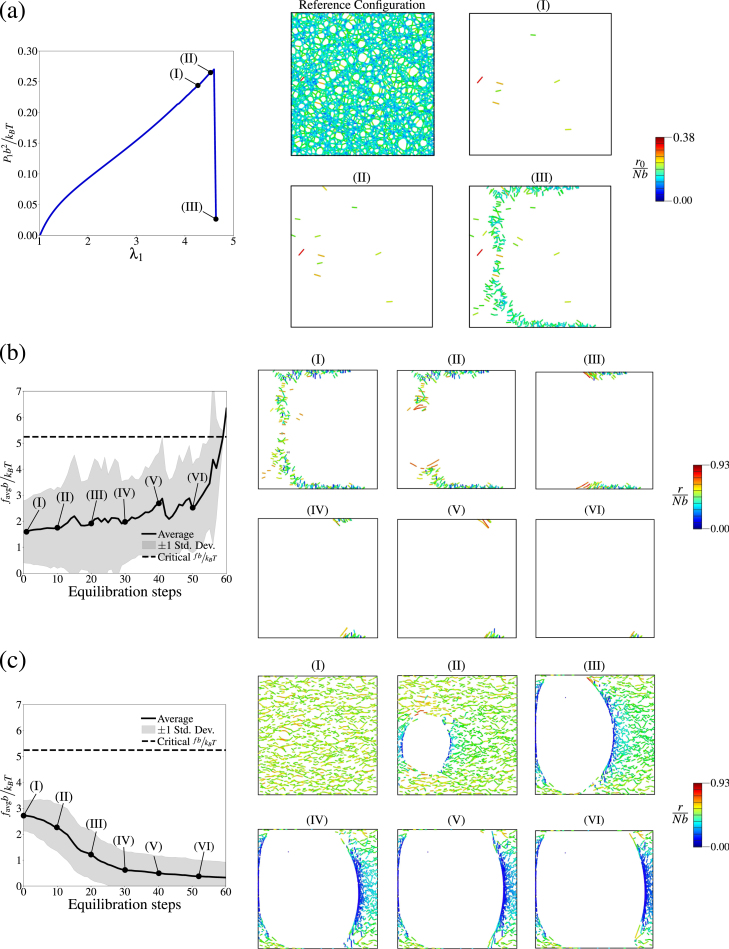
(a) Uniaxial tension response of a 2D DN with $\nu_{0}b^{2}=3\times 10^{-2}$, average pre-stretch ${\overset{¯}{\lambda }}_{0}\approx 1$, chain length N=100, and scission threshold η=0.8. Snapshots show the broken chains at selected points along the stress–stretch curve, coloured by their normalised initial end-to-end distance $\frac{r_{0}}{Nb}$. (b)–(c) Evolution of the average normalised force $\frac{f_{\mathrm{avg}}b}{k_{B}T}$ for the chains (b) that eventually break during the sharp stress drop, and (c) the top 10% most loaded chains at the onset of the stress drop. Snapshots show these chains mapped to the reference configuration and coloured by their current normalised elongation $\frac{r}{Nb}$.

## Data Availability

Data will be made available on request.

## Appendix A 2D example

The failure process is more easily visualised in a two-dimensional discrete network. Fig. 15(a) shows the uniaxial tension response of a representative 2D DN with chain density $\nu_{0}b^{2}=3\times 10^{-2}$, average pre-stretch ${\overset{¯}{\lambda }}_{0}\approx 1$, chain length N=100, and scission threshold η=0.8. Snapshots of the network at selected points along the nominal stress–stretch curve are also shown, where broken chains are coloured by their normalised initial end-to-end distance $\frac{r_{0}}{Nb}$. Different from the 3D case, loss of connectivity is reached immediately after the stress drop. The snapshots preceding the stress drop (I and II) show that the first scissions occur preferentially in chains with large initial end-to-end distances. The final snapshot (III) reveals that the stress drop is again associated with localised failure of the network, involving a chain scission cascade triggered by force redistribution within a spatially localised cluster of chains.

To gain further insight into the micromechanisms underlying this catastrophic event, Fig. 15(b) and (c) show the evolution of the average normalised chain force $\frac{f_{\mathrm{avg}}b}{k_{B}T}$ during the stress drop for two populations of chains: (i) the chains that eventually break during the drop (Fig. 15(b)) and (ii) the top 10% most loaded chains at the onset of the drop (Fig. 15(c)), together with snapshots of the corresponding chains mapped to the reference configuration and coloured by their current normalised elongation $\frac{r}{Nb}$. In these snapshots, chains are removed once they are broken. Consistent with the 3D results, Fig. 15(b) shows that most chains that ultimately break are initially below the scission threshold at the onset of the stress drop (dashed line). Their average force increases during the equilibration process, indicating that scission-induced force redistribution drives sub-critical chains to failure and sustains the cascade. Fig. 15(c) shows that the net effect of force redistribution is an overall reduction in the average force in the 10% most loaded chains. The snapshots also reveal that the 10% most loaded chains at the onset of the drop are homogeneously distributed across the domain. The first scissions occurring during the drop create a cavity in the network, around which force redistribution is particularly intense. This suggests that regions of locally reduced chain density can act as preferential sites for damage localisation and failure initiation.

## Appendix B Extended damage microsphere models

### B.1 Derivations

We provide derivation details for the extended microsphere models accounting for chain scission, starting from the free energy given in Eq. (16). Under the assumption of affine deformation, the initial orientation of a chain $n_{0}$ is mapped by the deformation gradient F to its current orientation as $n=Fn_{0}$. The chain stretch $\lambda_{c}=r/r_{0}$ can be expressed as $\lambda_{c}=\|Fn_{0}\|$. The network contribution $P^{e}$ to the macroscopic stress P (Eq. (7)) is obtained as $P^{e}=\frac{\partial \psi^{e}}{\partial F}$, giving:

(B.1) $$P^{e}=\nu_{0}\int_{r_{0}}p_{r_{0}}\left(r_{0}\right)\iint_{S_{u}}H\left(t,r\left(\tau \right)\right)\frac{fr_{0}}{\lambda_{c}}F\left(n_{0}⊗n_{0}\right)dS_{u}dr_{0}.$$

In the non-affine model, we follow Mulderrig et al. (2021) and postulate that the orientations of the chains in the deformed configuration should minimise the free energy of the network under a prescribed deformation gradient, subjected to the following kinematic constraint:

(B.2) $$\int_{r_{0}}p_{r_{0}}\left(r_{0}\right)\frac{r_{0}^{2}}{{\overset{¯}{r}}_{0}^{2}}\iint_{S_{u}}H\left(t,r\left(\tau \right)\right)Fn_{0}⊗n_{0}dS_{u}dr_{0}\;=\int_{r_{0}}p_{r_{0}}\left(r_{0}\right)\frac{r_{0}^{2}}{{\overset{¯}{r}}_{0}^{2}}\iint_{S_{u}}H\left(t\right)n⊗n_{0}dS_{u}dr_{0},$$

where ${\overset{¯}{r}}_{0}^{2}=\int_{r_{0}}p_{r_{0}}\left(r_{0}\right)r_{0}^{2}dr_{0}$. Compared to Mulderrig et al. (2021), the damage microsphere model is here extended to account for an initial distribution of chain end-to-end distances. The weighting factors $r_{0}^{2}/{\overset{¯}{r}}_{0}^{2}$ were included in the integrals over $r_{0}$ to ensure that the model recovers the Gaussian chain behaviour at small deformations, see Mangas Araujo et al. (2024). When the chain initial end-to-end distance is assumed uniform, and in the absence of damage, the kinematic constraint reduces to:

(B.3) $$\frac{1}{3}F=\iint_{S_{u}}F_{m}dS_{u},$$

where $F_{m}=n⊗n_{0}$ represents a microscopic deformation gradient. In the general case, the distribution of deformed orientations is obtained by solving the minimum problem:

(B.4) $$\psi^{e}=\underset{n,\pi }{\inf}\left\{\;\nu_{0}\int_{r_{0}}p_{r_{0}}\left(r_{0}\right)\iint_{S_{u}}H\left(t,r\left(\tau \right)\right)w\left(r\right)\;dS_{u}\;dr_{0}\;+\pi :\left[\int_{r_{0}}p_{r_{0}}\left(r_{0}\right)\frac{r_{0}^{2}}{{\overset{¯}{r}}_{0}^{2}}\iint_{S_{u}}H\left(t,r\left(\tau \right)\right)Fn_{0}⊗n_{0}\;dS_{u}dr_{0}\;-\int_{r_{0}}p_{r_{0}}\left(r_{0}\right)\frac{r_{0}^{2}}{{\overset{¯}{r}}_{0}^{2}}\iint_{S_{u}}H\left(t,r\left(\tau \right)\right)n⊗n_{0}\;dS_{u}dr_{0}\right]\right\},$$

where π is the Lagrange multiplier enforcing the constraint (B.2). The stationary condition with respect to n gives, for each $r_{0}$:

(B.5) $$f=\frac{r_{0}}{\nu_{0}{\overset{¯}{r}}_{0}^{2}}\pi n_{0},$$

which can be inserted back into Eq. (B.2) to yield an implicit equation for the multiplier π. Taking the stationary condition into account, the network contribution $P^{e}$ to the macroscopic stress P is obtained as:

(B.6) $$P^{e}=\int_{r_{0}}p_{r_{0}}\left(r_{0}\right)\frac{r_{0}^{2}}{{\overset{¯}{r}}_{0}^{2}}\iint_{S_{u}}H\left(t\right)\pi n_{0}⊗n_{0}dS_{u}dr_{0}.$$

### B.2 Implementation of the fully-relaxed model

The fully-relaxed model was implemented using a staggered approach that mimics the quasi-static procedure employed in the DN simulations (see Fig. 1(b)). The end-to-end distance distribution $p_{r_{0}}\left(r_{0}\right)$ was directly extracted from a representative DN. This distribution was then discretised into $n_{r_{0}}$ points. In practice, $n_{r_{0}}=1000$ points were used. For each value of $r_{0}$ in the discretisation, we associated a list $I\left(r_{0}\right)$ encoding the history-dependent indicator function H(t) evaluated over orientations on the unit sphere. All chains are assumed to be intact at initialisation. Integration over the unit sphere was carried out using 900 integration points (Fliege and Maier, 1999).

At each macroscopic stretch increment, the implicit Eq. (B.2) for the multiplier π is solved using the list $I\left(r_{0}\right)$ from the previous increment. Once π is determined, the distribution of deformed orientations is evaluated via Eq. (B.5), and $I\left(r_{0}\right)$ is updated accordingly to reflect newly broken chains. If chain scission occurred during this update, the implicit equation for π is resolved with the updated $I\left(r_{0}\right)$, and the cycle repeats: a new π is computed, leading to a new distribution of deformed orientation and a new list $I\left(r_{0}\right)$. This iterative procedure continues until no additional cleavage is detected.
